# Whole breast and regional nodal irradiation in prone versus supine position in left sided breast cancer

**DOI:** 10.1186/s13014-017-0828-6

**Published:** 2017-05-26

**Authors:** Pieter Deseyne, Bruno Speleers, Wilfried De Neve, Bert Boute, Leen Paelinck, Tom Van Hoof, Joris Van de Velde, Annick Van Greveling, Chris Monten, Giselle Post, Herman Depypere, Liv Veldeman

**Affiliations:** 1 Department of Radiation Oncology, Ghent University Hospital, De Pintelaan 185, Ghent, B-9000 Belgium; 2 Department of Radiotherapy and Experimental Cancer Research, Faculty of Medicine and Health Sciences, Ghent University, De Pintelaan 185, Ghent, B-9000 Belgium; 3 Industrial Design Centre - Department of Industrial Systems Engineering and Product Design (EA18), Faculty of Engineering and Architecture, Ghent University, Campus Kortrijk, Graaf Karel de Goedelaan 5, Kortrijk, B-8500 Belgium; 4 Department of Anatomy, Faculty of Medicine and Health Sciences, Ghent University, De Pintelaan 185, Ghent, B-9000 Belgium; 5 Department of Obstetrics and Gynaecology, Ghent University Hospital, De Pintelaan 185, Ghent, B-9000 Belgium; 6 Department of Uro-gynaecology, Ghent University, De Pintelaan 185, Ghent, B-9000 Belgium

**Keywords:** Adjuvant radiotherapy, Breast irradiation, Regional nodal irradiation, Breast cancer, Prone position, Prone crawl position, Dosimetry, Organs at risk, VMAT

## Abstract

**Background:**

Prone whole breast irradiation (WBI) leads to reduced heart and lung doses in breast cancer patients receiving adjuvant radiotherapy. In this feasibility trial, we investigated the prone position for whole breast + lymph node irradiation (WB + LNI).

**Methods:**

A new support device was developed for optimal target coverage, on which patients are positioned in a position resembling a phase from the crawl swimming technique (prone crawl position). Five left sided breast cancer patients were included and simulated in supine and prone position. For each patient, a treatment plan was made in prone and supine position for WB + LNI to the whole axilla and the unoperated part of the axilla. Patients served as their own controls for comparing dosimetry of target volumes and organs at risk (OAR) in prone versus in supine position.

**Results:**

Target volume coverage differed only slightly between prone and supine position. Doses were significantly reduced (*P* < 0.05) in prone position for ipsilateral lung (Dmean, D2, V5, V10, V20, V30), contralateral lung (Dmean, D2), contralateral breast (Dmean, D2 and for total axillary WB + LNI also V5), thyroid (Dmean, D2, V5, V10, V20, V30), oesophagus (Dmean and for partial axillary WB + LNI also D2 and V5), skin (D2 and for partial axillary WB + LNI V105 and V107). There were no significant differences for heart and humeral head doses.

**Conclusions:**

Prone crawl position in WB + LNI allows for good breast and nodal target coverage with better sparing of ipsilateral lung, thyroid, contralateral breast, contralateral lung and oesophagus when compared to supine position. There is no difference in heart and humeral head doses.

**Trial registration:**

No trial registration was performed because there were no therapeutic interventions.

## Background

Locoregional irradiation in breast cancer decreases recurrences and breast cancer mortality in patients with positive lymph nodes [1–3]. Traditionally, locoregional irradiation has been administered by using two high tangential fields with the patient in supine position. These techniques have demonstrated to insufficiently dose the axillary lymph node regions [4, 5]. Separate boost fields could be added to the axilla and the supraclavicular region to improve coverage of these areas, at the cost of higher OAR doses [6]. Newer techniques such as intensity modulated radiotherapy (IMRT) and volumetric modulated arc therapy (VMAT) [7–9] have led to dosimetrical benefits, including better sparing of organs at risk (OARs) and better dose homogeneity, but with higher volumes of healthy tissue receiving low dose.

Aside from these techniques, other ways to decrease the dose to the heart and lungs have been implemented in whole breast irradiation (WBI), such as the prone position. A comparative dosimetrical study shows that in prone position, even simple wedged tangential fields improve dose to the ipsilateral lung when compared to any of the more advanced static supine techniques used [8]. Other authors agree that the prone position allows for better sparing of the ipsilateral lung [8, 10, 11]. In left sided breast cancer, the dose to the heart is also decreased in the majority of patients by using the prone position, but published results show this might not be the case for all patients [8, 10–13]. Therefore, additional efforts were made to lower the dose to the heart by using breath-hold or gating techniques [14–17].

Although some authors report dosimetrical data on regional nodal irradiation in prone position [18, 19], this indication has not seen the same shift towards prone irradiation as in WBI, and few data are available for clinical implementation [20].

Commercially available devices for prone breast irradiation typically support the patient with both arms elevated alongside the head. One of the major disadvantages of treating patients in this position, however, is that the support material itself obstructs the anterior beam access to the regional lymph nodes, which creates a bolus effect with possible associated skin toxicities. The use of other beam directions increases the trajectory through healthy tissues and therefore increases doses to OARs [18, 21].

The aim to obtain unobstructed beam access to the breast and its regional lymph nodes while maintaining the benefits of the prone position regarding dose to the organs at risk (OAR) led to the investigation of a new patient prone position and its specifically adapted patient support surface. In this prone position, the arm of the patient at the treated side is positioned alongside the body, the arm at the contralateral side above the head. This study presents data on the feasibility of whole-breast irradiation with lymph node irradiation (WB + LNI) in this position.

## Methods

### Prone crawl positioning device

**Fig. 1:**
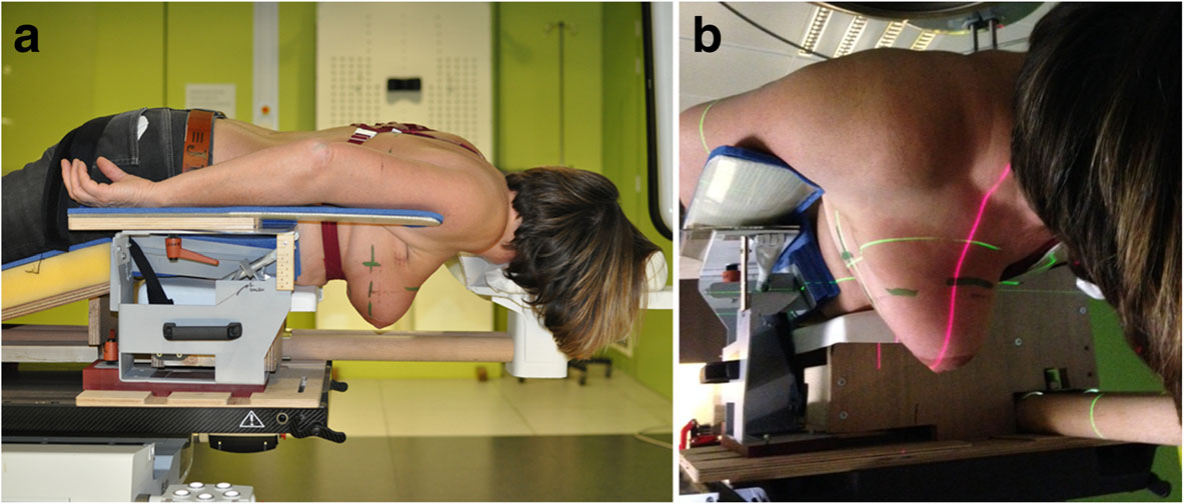
Patient setup on a prototype of the crawl breast couch. **a** The ipsilateral arm is positioned on a support along the waist. The head is turned away from the ispilateral side towards the contralateral arm, which is extended along the head. **b** Red floor laser beam projected on the breast

To overcome the disadvantages of conventional prone positioning aids, our team developed a prototype with a surface not directly supporting the target regions for WB + LNI such as the axillary, periclavicular or ipsilateral internal mammary nodal regions or the treated breast [Fig. 1]. The prototype does not rest entirely on the treatment couch, as is usually the case in prone positioning devices, further called breast *boards*. The device is anchored on the caudal part of the treatment couch, while the cranial part of the couch is removed to allow for an overhanging position of the prototype without support from the treatment unit couch [Fig. 1]. This design – further called a breast *couch –* allows the use of a floor laser for patient positioning, which can help to reduce lateral setup errors. The patient is positioned on the breast couch with the ipsilateral arm next to the body and the contralateral arm elevated alongside the head. This improves comfort for the operated axilla. Using the ipsilateral arm support as a waist support improved stability, by preventing patients from sliding down from the wedge. We refer to this position as the crawl position, because it resembles a phase of the crawl swimming technique.

### Patient selection

Five left sided breast cancer patients with invasive carcinoma of the breast and pathologically confirmed positive lymph node status were included in our study. All patients underwent lumpectomy and axillary clearance followed by adjuvant WB + LNI in supine position. Informed consent was obtained before simulation for each patient. The study was approved by the local ethics board.

### Simulation

Patients were positioned and scanned in both the supine and prone crawl position. In supine position, patients were positioned using a Posirest arm support (Civco Medical Solutions, Kalone, Iowa, United States) with both arms elevated above the head. Opaque wires were positioned around the contours of both breasts, as well as on the tumorectomy scar. Patients were administered IV iodine contrast prior to scanning. After supine simulation, patients were positioned on the crawl breast couch for prone simulation. An opaque wire was only placed around the ipsilateral breast and surgical incision. The contralateral breast was pulled laterally, away from the ipsilateral breast to increase distance to the irradiated area and fixated in a unilateral bra [22]. For both positions, 5 mm slice thickness images were acquired on a Siemens SOMATOM definition AS computed tomography-scan (Siemens Healthineers, Erlangen, Germany), starting at the vertex and caudally including the whole breast and both lungs.

### Target volume delineation

All contours were delineated on the Pinnacle^3^ 9.8 treatment planning system (Philips Healthcare, Fitchburg, Wisconsin, United States). In supine position, clinical target volumes for whole breast irradiation (CTV_WBI) and lymph node irradiation (CTV_LNI) were defined according to the contouring guidelines as proposed by the ESTRO and PROCAB groups [23–26]. As there are no generally accepted guidelines for delineation in prone position, we performed delineation of CTV_WBI and CTV_LNI using extrapolation from the guidelines described above.

For each patient, two sets of CTV_LNI were delineated: the whole axilla (level I-II-III-IV) and the partial axilla (level II-III-IV, i.e. the undissected axillary regions after surgical clearance). Internal mammary nodes were not contoured or included in any of the treatment volumes. Planning target volume was obtained by performing an isometric expansion of CTV_WBI and CTV_LNI by 5 mm and 3 mm, respectively, thereby creating PTV_WBI and PTV_LNI. For PTV_WBI no expansion in the medial direction was done to avoid irradiation of the contralateral breast. Planning optimization structures (PTV_WBI_opt and PTV_LNI_opt) were created to account for dose buildup underneath the skin. The structures were made by retracting PTV_WBI and PTV_LNI 8 mm away from the skin and excluding the heart, lung and contralateral breast, all with an expansion of 8 mm, adding the delineated clinical target volumes. Because these optimization structures are the structures used in planning, they will be referred to from hereon as the PTV_WBI and PTV_LNI, unless specifically stated otherwise. All delineations were performed by the same experienced radiation oncologist.

### Organs at risk (OAR) delineation

The heart was delineated in accordance with guidelines proposed by Feng et al. [27]. Left and right lungs were contoured separately using automatic segmentation by Hounsfield units options provided in Pinnacle^3^ 9.8 with threshold 800–4096. The contralateral breast was delineated up to the skin. The thyroid was manually delineated where visible, as was the oesophagus, starting cranially from the inferior margin of the cricoid and ending inferiorly at the gastro-oesophageal margin. The humeral head was delineated from the most proximal part to the lower border of the tuberculum maius. The skin was defined as the scanned volume of the patient.

### Treatment planning

**Fig. 2:**
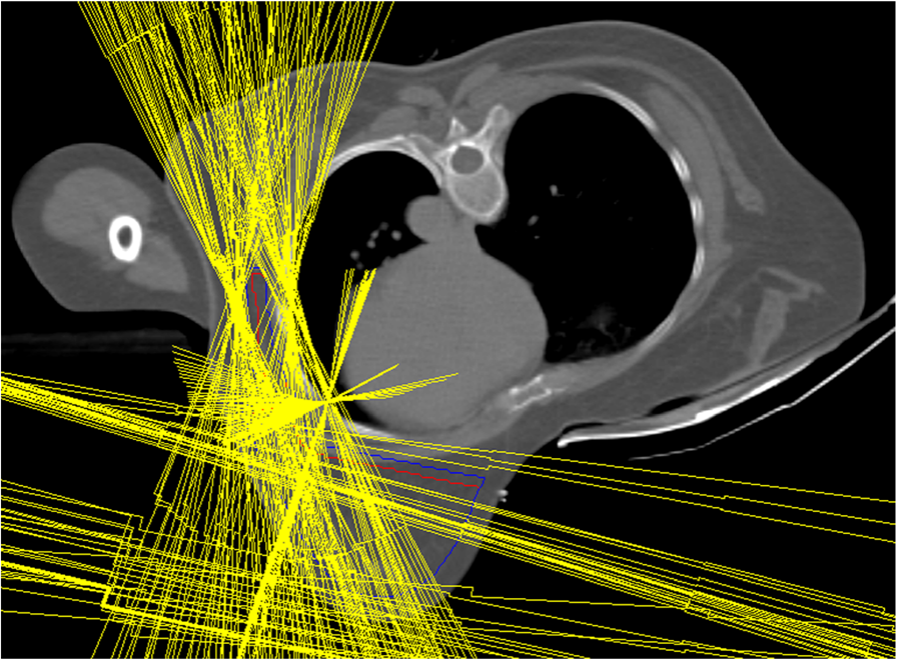
Illustration of the coplanar multiple overlying partial arc VMAT technique. The *yellow lines* indicate beam apertures of the different used beam angles, avoiding OAR such as the heart, lung and the ipsilateral arm

All treatment plans were made by the same treatment planner. For every patient, four plans were created: WB + LNI to the whole axilla in prone and supine position, and WB + LNI to the partial axilla in prone and supine position. A coplanar multiple overlying partial arc VMAT technique was used to avoid excessive low-dose spread to the organs at risk [Fig. 2]. All plans were optimized using GRATIS (Sherouse-on-Hudson Medical Physics, High Falls, New York, United States) software with in-house developed modifications. The final dose calculation was performed using the collapsed cone convolution dose computation engine in Pinnacle^3^ 9.8.

The dosimetric comparisons were made for 6 MV energy photon beams for an Elekta Synergy linear accelerator (Elekta, Crawley, UK), equipped with an MLC with a leaf-width of 5 mm.

### Dose prescription and measured dose statistics

A median dose of 40.05 Gy in 15 fractions of 2.67 Gy was prescribed to the PTV_WBI and PTV_LNI. The objective was to cover 95% of the target volume with at least 95% of the prescribed dose (i.e. 38 Gy), with no more than 5% receiving 105% of the prescribed dose (i.e. 42 Gy). Dose heterogeneity was evaluated according to the formula below, suggested in ICRU report 83 [28], where D2 and D98 are used as substitutes of the maximal and minimal point dose in a delineated volume, because the latter two parameters are subject to variations in dose-calculation parameters and therefore less reliable.

$$ H=\frac{D2- D98}{D50} $$

**Fig. 3:**
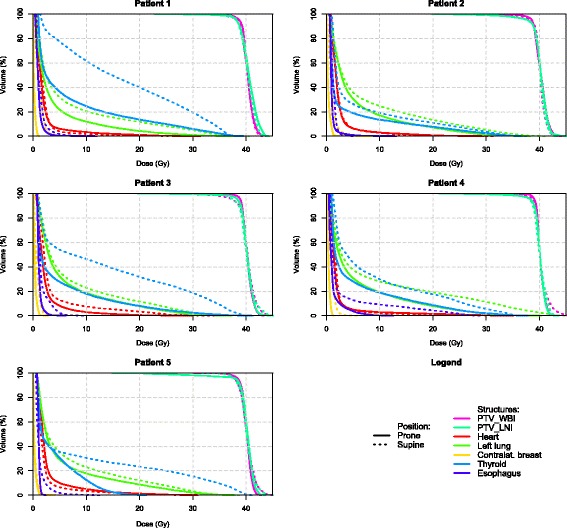
Individual DVH’s for each patient in WB + LNI to the whole axilla

**Fig. 4:**
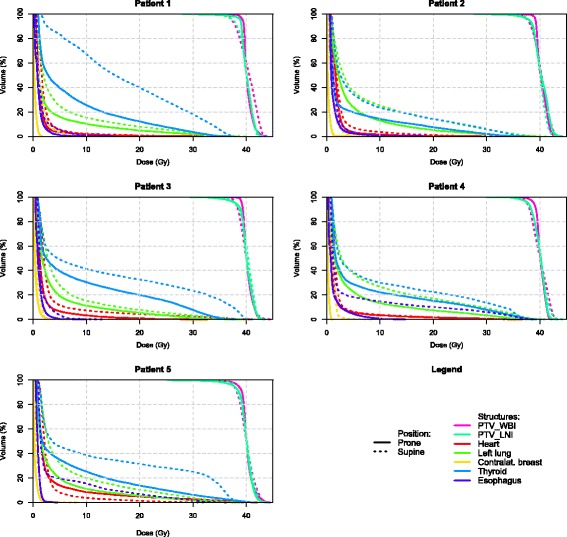
Individual DVH’s for each patient in WB + LNI to the partial axilla

Dose statistics are referred to as D*n* (the minimal dose delivered to *n* % of the volume) or V*n* (the volume percentage receiving ≥ *n* Gy). We generated dose-volume histograms (DVH) for all patients, showing dosimetry for prone and supine treatment positions in either whole or partial axillary LNI [Figs. 3 and 4]. All patients required a boost to the tumor bed, but because the location varied for each patient, we did not include the boost dose in our analysis, so as to preserve uniformity in our small patient group. Hereby we were able to eliminate the risk of bias when interpreting the dosimetry results and attributing changes caused by a boost dose to the way the patient was positioned and vice versa.

### Data analysis

Data was collected and analysed in R. We used non-parametric testing for data analysis, because of the nature of the data and inapplicability of the central limit theorem in this group with limited sample size. Data on PTV’s an OAR’s were compared and analysed per treatment position and treatment plan (whole or partial axillary LNI). After visual data inspection, we used a one-sided Wilcoxon signed ranks test for related samples with a significance level of < 0.05.

## Results

Dose statistics for targets and OAR’s are provided in Tables 1 and 2. DVH’s are shown for both whole and partial axillary LNI in Figs. 3 and 4. Reported results apply to both axillary and partial axillary LNI unless specified otherwise.

**Table 1:** Target volume dose parameters

Target Volumes		Axilla prone	Axilla supine	Significance	Partial axilla prone	Partial axilla supine	Significance
CTV_WBI							
	Volume (ml)	420 ± 165	477 ± 174	*P* < 0.05	420 ± 165	477 ± 174	*P* < 0.05
	Dmean (Gy)	40.26 ± 0.09	40.34 ± 0.12	-	40.20 ± 0.10	40.27 ± 0.33	-
	D02 (Gy)	42.12 ± 0.38	43.12 ± 0.80	-	42.16 ± 0.44	42.80 ± 0.35	*P* < 0.05
	D05 (Gy)	41.74 ± 0.29	42.40 ± 0.49	-	41.72 ± 0.24	42.43 ± 0.41	*P* < 0.05
	D50 (Gy)	40.21 ± 0.10	40.24 ± 0.16	-	40.03 ± 0.06	40.31 ± 0.41	-
	D95 (Gy)	38.98 ± 0.16	38.63 ± 0.45	-	39.13 ± 0.24	38.00 ± 0.18	*P* < 0.05
	D98 (Gy)	38.47 ± 0.24	38.01 ± 0.58	*P* < 0.05	38.72 ± 0.41	37.45 ± 0.23	*P* < 0.05
	Heterogeneity	0.09 ± 0.01	0.13 ± 0.03	-	0.09 ± 0.02	0.13 ± 0.01	*P* < 0.05
PTV_WBI							
	Volume (ml)	483 ± 176	560 ± 204	*P* < 0.05	483 ± 176	560 ± 204	*P* < 0.05
	Dmean	40.23 ± 0.09	40.19 ± 0.06	-	40.23 ± 0.07	40.15 ± 0.16	-
	D02	42.26 ± 0.42	43.06 ± 0.75	-	42.34 ± 0.51	42.86 ± 0.19	-
	D05	41.85 ± 0.32	42.34 ± 0.46	-	41.90 ± 0.30	42.46 ± 0.30	*P* < 0.05
	D50	40.21 ± 0.10	40.18 ± 0.10	-	40.05 ± 0.02	40.19 ± 0.25	-
	D95	38.76 ± 0.23	38.20 ± 0.50	*P* < 0.05	39.02 ± 0.31	37.68 ± 0.25	*P* < 0.05
	D98	37.91 ± 0.48	36.54 ± 0.96	*P* < 0.05	38.45 ± 0.62	37.02 ± 0.30	*P* < 0.05
	Heterogeneity	0.11 ± 0.02	0.16 ± 0.03	*P* < 0.05	0.10 ± 0.02	0.15 ± 0.01	*P* < 0.05
CTV_LNI							
	Volume (ml)	114 ± 31	89 ± 19	*P* < 0.05	51 ± 18	28 ± 3	*P* < 0.05
	Dmean	40.30 ± 0.32	40.28 ± 0.13	-	40.12 ± 0.17	40.26 ± 0.15	-
	D02	42.57 ± 0.55	42.94 ± 0.73	-	42.39 ± 0.34	42.32 ± 0.60	-
	D05	42.09 ± 0.58	42.42 ± 0.55	-	41.99 ± 0.29	42.01 ± 0.19	-
	D50	40.40 ± 0.27	40.23 ± 0.15	-	40.16 ± 0.13	40.33 ± 0.13	-
	D95	38.42 ± 0.27	38.45 ± 0.28	-	37.99 ± 0.63	38.26 ± 0.36	-
	D98	36.11 ± 2.07	37.47 ± 0.57	-	36.72 ± 1.22	37.54 ± 0.48	-
	Heterogeneity	0.16 ± 0.05	0.14 ± 0.02	-	0.14 ± 0.03	0.12 ± 0.02	-
PTV_LNI							
	Volume (ml)	192 ± 51	152 ± 30	*P* < 0.05	82 ± 34	51 ± 6	-
	Dmean	40.01 ± 0.35	40.07 ± 0.17	-	39.84 ± 0.31	40.04 ± 0.10	-
	D02	42.55 ± 0.56	42.87 ± 0.62	-	42.31 ± 0.37	42.32 ± 0.31	-
	D05	42.08 ± 0.56	42.27 ± 0.41	-	41.89 ± 0.32	41.96 ± 0.22	-
	D50	40.27 ± 0.19	40.11 ± 0.15	*P* < 0.05	40.03 ± 0.10	40.15 ± 0.10	-
	D95	37.02 ± 1.47	37.81 ± 0.32	-	36.86 ± 1.43	37.67 ± 0.22	-
	D98	33.12 ± 4.25	36.11 ± 1.24	*P* < 0.05	34.40 ± 2.42	36.69 ± 0.39	-
	Heterogeneity	0.23 ± 0.10	0.17 ± 0.03	-	0.20 ± 0.06	0.14 ± 0.01	-
PTV_LNI_opt							
	Volume (ml)	177 ± 50	134 ± 31	*P* < 0.05	75 ± 32	50 ± 6	-
	Dmean	40.15 ± 0.24	40.11 ± 0.14	-	40.07 ± 0.13	40.06 ± 0.11	-
	D02	42.53 ± 0.54	42.88 ± 0.65	-	42.33 ± 0.35	42.32 ± 0.31	-
	D05	42.06 ± 0.55	42.32 ± 0.47	-	41.93 ± 0.30	41.97 ± 0.22	-
	D50	40.29 ± 0.17	40.13 ± 0.13	*P* < 0.05	40.12 ± 0.09	40.16 ± 0.11	-
	D95	38.18 ± 0.26	37.90 ± 0.31	-	38.10 ± 0.47	37.76 ± 0.19	-
	D98	34.86 ± 2.90	36.32 ± 1.18	-	36.46 ± 0.73	36.78 ± 0.38	-
	Heterogeneity	0.19 ± 0.07	0.16 ± 0.03	-	0.15 ± 0.02	0.14 ± 0.01	-

Data are presented as group average ± standard deviation

**Table 2:** OAR dose volume parameters

Organs at Risk		Axilla prone	Axilla supine	Significance	Partial axilla prone	Partial axilla supine	Significance
Heart							
	Volume (ml)	631 ± 109	649 ± 141	-	631 ± 109	649 ± 141	-
	Dmean (Gy)	2.38 ± 0.28	2.52 ± 0.59	-	2.24 ± 0.85	2.83 ± 0.61	-
	D02 (Gy)	14.83 ± 1.64	14.02 ± 5.60	-	16.18 ± 10.22	18.75 ± 7.94	-
	V5 (%)	7.10 ± 1.93	6.83 ± 3.56	-	6.60 ± 4.32	7.79 ± 2.32	-
	V10 (%)	3.47 ± 0.92	3.51 ± 2.35	-	3.69 ± 2.86	4.12 ± 1.91	-
	V20 (%)	1.04 ± 0.39	1.23 ± 1.20	-	1.60 ± 1.86	1.90 ± 1.45	-
	V30 (%)	0.10 ± 0.18	0.06 ± 0.05	-	0.66 ± 1.10	0.64 ± 1.02	-
Ipsilateral lung							
	Volume (ml)	1240 ± 364	1158 ± 290	-	1240 ± 364	1158 ± 290	-
	Dmean	5.52 ± 0.73	7.51 ± 1.03	*P* < 0.05	4.22 ± 0.53	6.82 ± 1.35	*P* < 0.05
	D02	28.44 ± 1.16	34.28 ± 4.05	*P* < 0.05	29.71 ± 1.83	34.19 ± 1.93	*P* < 0.05
	V5	28.85 ± 1.62	35.35 ± 2.11	*P* < 0.05	20.32 ± 3.09	31.81 ± 5.45	*P* < 0.05
	V10	16.91 ± 2.94	23.80 ± 2.77	*P* < 0.05	11.82 ± 1.30	20.54 ± 5.39	*P* < 0.05
	V20	7.13 ± 1.69	13.59 ± 3.22	*P* < 0.05	5.65 ± 1.03	11.47 ± 4.03	*P* < 0.05
	V30	1.50 ± 0.47	5.49 ± 3.46	*P* < 0.05	1.94 ± 0.80	5.01 ± 2.54	*P* < 0.05
Contralateral lung							
	Volume (ml)	1536 ± 303	1458 ± 318	-	1538 ± 303	1458 ± 318	-
	Dmean	0.35 ± 0.04	0.60 ± 0.09	*P* < 0.05	0.31 ± 0.05	0.84 ± 0.25	*P* < 0.05
	D02	0.93 ± 0.06	1.93 ± 0.21	*P* < 0.05	0.90 ± 0.10	4.28 ± 2.95	*P* < 0.05
	V5	0.00 ± 0.00	0.04 ± 0.07	-	0.00 ± 0.00	1.30 ± 1.67	-
	V10	0.00 ± 0.00	0.01 ± 0.03	-	0.00 ± 0.00	0.45 ± 0.58	-
	V20	0.00 ± 0.00	0.00 ± 0.00	-	0.00 ± 0.00	0.01 ± 0.01	-
	V30	0.00 ± 0.00	0.00 ± 0.00	-	0.00 ± 0.00	0.00 ± 0.00	-
Contralateral breast							
	Volume (ml)	703 ± 235	665 ± 232	-	703 ± 235	665 ± 232	-
	Dmean	0.33 ± 0.07	0.83 ± 0.30	*P* < 0.05	0.36 ± 0.10	1.01 ± 0.28	*P* < 0.05
	D02	1.07 ± 0.16	2.54 ± 0.84	*P* < 0.05	1.30 ± 0.45	3.31 ± 1.70	*P* < 0.05
	V5	0.02 ± 0.05	0.20 ± 0.23	*P* < 0.05	0.03 ± 0.04	0.86 ± 1.45	-
	V10	0.00 ± 0.00	0.00 ± 0.00	-	0.00 ± 0.00	0.00 ± 0.00	-
	V20	0.00 ± 0.00	0.00 ± 0.00	-	0.00 ± 0.00	0.00 ± 0.00	-
	V30	0.00 ± 0.00	0.00 ± 0.00	-	0.00 ± 0.00	0.00 ± 0.00	-
Thyroid							
	Volume (ml)	22 ± 14	19 ± 15	-	22 ± 14	19 ± 15	-
	Dmean	5.38 ± 1.37	10.96 ± 4.11	*P* < 0.05	7.05 ± 1.64	12.21 ± 3.69	*P* < 0.05
	D02	18.63 ± 7.53	36.04 ± 3.00	*P* < 0.05	33.91 ± 3.00	36.83 ± 1.88	*P* < 0.05
	V5	28.29 ± 6.33	47.29 ± 19.64	*P* < 0.05	32.55 ± 8.44	50.32 ± 19.46	*P* < 0.05
	V10	17.48 ± 4.82	37.49 ± 16.60	*P* < 0.05	23.55 ± 5.95	40.15 ± 16.76	*P* < 0.05
	V20	7.64 ± 4.82	24.66 ± 11.42	*P* < 0.05	13.73 ± 4.58	28.07 ± 9.88	*P* < 0.05
	V30	2.57 ± 2.13	12.57 ± 7.46	*P* < 0.05	5.47 ± 2.63	17.21 ± 7.72	*P* < 0.05
Oesophagus							
	Volume (ml)	35 ± 16	27 ± 12	*P* < 0.05	35 ± 16	27 ± 12	*P* < 0.05
	Dmean	1.11 ± 0.09	1.74 ± 0.78	*P* < 0.05	1.12 ± 0.14	2.88 ± 1.75	*P* < 0.05
	D02	3.36 ± 1.80	8.33 ± 8.96	-	4.49 ± 2.09	16.92 ± 14.49	*P* < 0.05
	V5	1.00 ± 1.53	3.73 ± 5.14	-	1.70 ± 1.83	9.91 ± 8.21	*P* < 0.05
	V10	0.06 ± 0.08	1.85 ± 4.03	-	0.26 ± 0.32	6.25 ± 8.12	-
	V20	0.00 ± 0.00	0.87 ± 1.94	-	0.00 ± 0.00	3.42 ± 4.83	-
	V30	0.00 ± 0.00	0.00 ± 0.00	-	0.00 ± 0.00	1.53 ± 2.59	-
Humeral Head							
	Volume (ml)	57 ± 15	63 ± 23	-	57 ± 15	63 ± 23	-
	Dmean	9.75 ± 3.75	7.74 ± 2.94	-	9.85 ± 3.64	10.69 ± 3.33	-
	D02	21.90 ± 1.49	25.08 ± 7.98	-	23.39 ± 2.10	27.22 ± 3.37	-
	V5	69.76 ± 22.06	47.14 ± 23.45	-	64.46 ± 20.25	56.82 ± 13.37	-
	V10	45.47 ± 31.68	23.87 ± 15.82	-	43.80 ± 25.85	45.89 ± 16.08	-
	V20	4.05 ± 1.50	8.68 ± 7.57	-	9.25 ± 6.17	19.60 ± 13.52	-
	V30	0.03 ± 0.06	1.98 ± 3.00	-	0.01 ± 0.03	1.37 ± 2.00	-
Skin							
	Volume (ml)	34353 ± 9812	22946 ± 4460	-	34353 ± 9812	22946 ± 4460	-
	D02	38.40 ± 3.61	40.25 ± 0.15	*P* < 0.05	37.19 ± 5.16	39.95 ± 0.54	*P* < 0.05
	V105 (%)	0.19 ± 0.14	0.30 ± 0.10	-	0.14 ± 0.10	0.38 ± 0.14	*P* < 0.05
	V105 (ml)	58.48 ± 37.01	68.86 ± 25.29	-	44.28 ± 36.22	91.48 ± 45.69	*P* < 0.05
	V107 (%)	0.06 ± 0.05	0.10 ± 0.05	-	0.03 ± 0.04	0.12 ± 0.09	*P* < 0.05
	V107 (ml)	17.85 ± 13.43	21.85 ± 11.43	-	9.77 ± 12.94	29.68 ± 27.28	*P* < 0.05

Data are presented as group average ± standard deviation

### Target volumes

There was a small, but statistically significant difference in CTV_WBI volume between both positions, i.e. 57 cc lower in prone than in supine position. In contrast, CTV_LNI volumes were significantly larger in prone position. Planning objectives for PTV_WBI and PTV_LNI were reached with minimal differences in D50, D95, D98, heterogeneity and hot spots between prone and supine positions.

### OAR’s

Significant differences were seen for ipsilateral lung dose on all measured DVH parameters, favouring the prone position. For the contralateral lung, a lower mean dose and D2 were observed in prone position, but differences in V5, V10, V20 or V30 were not significant. The contralateral breast showed decreased Dmean, D2 in prone as compared to supine position, as well as decreased V5 values in total axillary LNI for the prone position (*P* < 0.05). For the thyroid gland, Dmean, D2, V5, V10, V20 and V30 values were significantly lower in prone position (*P* < 0.05). Oesophageal Dmean was slightly higher in supine as compared to prone position, but there was also a volume difference, with larger volumes in prone position. Partial axillary LNI also showed a higher oesophageal D2 and V5 in supine position. For the skin, there was a higher D2 in the supine position in both LNI groups, as well as a higher V105 and V107 for partial axillary LNI (*P* < 0.05). There were no significant differences in DVH criteria for doses to the heart or humeral head. Aside from the oesophagus, there were no significant volume differences for OAR’s.

## Discussion

Prone WBI has led to improved dose homogeneity and reduced skin toxicity, while reducing the dose to the lung and heart [8, 10–13, 22, 29–31]. Due to a change in breast shape, path lengths are reduced and skin folds disappear, resulting in lower acute and late toxicity [22, 31].

Clinical data on prone WB + LNI is scarce. Shin et al. [20] have reported on LNI in prone, with concomitant WBI or chest wall irradiation. To our knowledge, this is the only clinical data reporting on prone LNI. Treatment in prone position showed significantly lower lung and heart doses as compared to another patient group treated to the same target regions in supine position. They reported no grade >2 acute dermatitis and only 4% of patients could not be treated in prone position. Long-term toxicity was limited, but median follow up was just under 3 years.

Gielda et al. [18] performed a study similar to our own, comparing WB + LNI planning in supine and prone position on 10 patients treated with tangential fields and one additional field to level IV. They observed no significant difference in target coverage, but the prone position resulted in lower dose to the ipsilateral lung and contralateral breast.

Kainz et al. [19] performed a feasibility trial comparing WB + LNI in prone position to previously reported supine tomotherapy plans. In left sided breast cancer, they showed lower heart and contralateral breast doses using the prone position.

The novelty of this study lies in the use of the prone crawl technique, with the ipsilateral arm alongside the body, for prone WB + LNI. The crawl breast couch allows beam access from underneath the patient without traversing support materials. To our knowledge, this is the first comparative study between prone and supine WB + LNI with each patient serving as their own control, while also aiming to provide adequate target coverage to all regional nodes with the exception of the internal mammary chain.

We were able to obtain treatment goals and constraints in all patients for either treatment position. The unoptimized PTV_LNI in axillary LNI shows a slight underdosage in prone when compared to supine position (D98 of 33.12 Gy compared to 36.11 Gy for axillary LNI and 34.40 Gy compared to 36.69 Gy for partial axillary LNI), although planning goals were obtained. This difference was significant for the PTV_LNI but not for the optimized PTV_LNI structure. We examined individual prone plans to check for regions of systematic underdosage, and compared these with the corresponding supine plans. We found that both plans have underdosage in the posterior inferomedial level IV region (where the internal mammary nodes start). This region is not easily treated without incurring significant dose in the lungs and thyroid, and therefore we allow a small underdosage to this region, as long as the D95 constraint is met. The difference between prone and supine plans, however, was that this underdosed region lies more inward because of the prone-lateral positioning, resulting in a larger part of this region being underdosed. A second difference is that using the delineation guidelines available, the PTV_LNI that was generated extended much closer to the skin in prone than supine position in the periclavicular region. This is why the PTV_LNI_opt does not show significantly more underdosage.

There was a significant volume difference in the target volumes between prone and supine position. For breast tissue, the prone position results in lower absolute volumes. It is possible that the crawl arm position does not stretch the breast out longitudinally as much as with elevated arms. Using a CT slice thickness of 5 mm, one or two slices less can lead to a significant volume difference.

The lymph node regions, however, have a larger absolute volume in prone position. Results were significantly different for whole axillary LNI, but not for partial axillary LNI, probably because the largest volume differences were seen in the low axillary region (level I), which is left out in partial axillary LNI. This might be due to the anatomy changes to the structures that define the lymph node regions in supine positioning, caused by an anterior shift of the breast in prone position. The breast “pulls” the lymph node regions anteriorly and elongates them in the anteroposterior axis, while posterior delineation margins, i.e. the subscapularis and latissimus dorsi muscles, remain more or less in place. The axillary levels III and IV are also more compressed in the supine position due to arm elevation compared to the crawl position with the arm alongside the body.

This study confirms the existing evidence of lowered ipsilateral lung doses in prone position, and adds to the smaller body of evidence that this benefit persists even when treating the regional lymph nodes [8, 10–12, 18, 20–22, 30, 32]. Exposure of the lung to radiotherapy increases the risk of second-primary lung cancer (SPLC) development and death [33–35]. Our data show a mean lung dose difference of 2.0 ± 1.0 Gy and 2.6 ± 0.9 Gy for axillary and periclavicular LNI, respectively, in benefit of the prone position. A dose response relationship with an increased RR for SPLC of 0.20 per incremental Gy of mean lung dose has been described by Inskip et al. [34]. Extrapolating these data to our population, the prone position could decrease the RR for ipsilateral lung cancer by 0.40 ± 0.20 and 0.52 ± 0.17 for axillary and periclavicular LNI respectively, and would decrease SPLC incidence from 6.76 ± 0.92 to 4.97 ± 0.66 and from 6.14 ± 1.21 to 3.80 ± 0.47 per year in 10,000 patients surviving 10 years or more [34]. A case–control study by Grantzau et al. showed that the rate of SPLC increased linearly with 8.5% per Gy in 5-year breast cancer treatment survivors [36]. All DVH parameters for ipsilateral lung in our study were significantly better in prone position. We could estimate a decrease in absolute excess risk of SPLC of up to about 50% in the highest lung dose regions in our whole axillary LNI patient group (291.38% ± 34.43% vs 241.74% ± 9.86%) and about 40% in our partial axillary LNI group (290.62% ± 16.41% vs 252.54% ± 15.56%) by using the prone position.

The reported lung doses are well below the suggested QUANTEC constraints [37] and the V5 constraint proposed by Jo et al. to limit the risk of radiation pneumonitis [38]. We also note that the contralateral lung receives less dose in prone than in supine position. However, in this low-dose region, the contribution of transmission and scatter dose is significant and treatment planning systems cannot reliably calculate these doses. Therefore, the relevance of these reduced calculated doses is unclear.

The dose to the contralateral breast is also reduced in prone position, likely because the crawl board allows for more optimal positioning of the contralateral breast, which is pulled away from the treatment fields and positioned in a shallow recess etched into the wedge. This further decreases the chance of the contralateral breast slipping off the wedge towards the treatment field. This decreased the D2 by about 50%, which could reduce the excess risk for contralateral breast cancer (CBC) at longer follow-up intervals [39–41].

The most pronounced dosimetrical difference between prone and supine treatment plans was observed for thyroid dose. In paediatric patients, a dose-response relationship has been described for secondary thyroid cancer [42], but these results have not been reproduced in breast cancer patients, where thyroid doses are generally lower. One Taiwanese study described an overall increased risk for thyroid cancer in breast cancer patients, regardless of irradiation [43]. They did not see an excess risk of thyroid cancer after radiotherapy for breast cancer. A meta-analysis by Grantzau and Overgaard failed to show an association between radiotherapy and secondary thyroid cancer in breast cancer patients [44]. However, a link has been demonstrated between radiation therapy and hypothyroidism. A meta-analysis by Vogelius et al. synthesizes multiple dose-responserelationships into one compounded model, indicating a 50% chance of developing hypothyroidism when the thyroid dose exceeded 45.15 Gy (95% CI 27.87 Gy – 62.43 Gy). The authors describe the dose-responserelationship as moderately steep, which indicates that incidence of hypothyroidism could be decreased by even a relatively small thyroid dose reduction [45]. Comparing the crawl with supine position, there was an important decrease in the thyroid dose using the crawl device for the low doses. This difference was even more pronounced for the high dose regions. The mean thyroid dose was halved, potentially indicating a substantial decrease in risk for radiation induced hypothyroidism.

For oesophageal doses, we noticed a decreased mean dose in prone position for axillary and partial axillary LNI, as well as lowered D2 and V5 in prone position for partial axillary LNI. As we also saw a significant increase in oesophageal volume in prone position, care needs to be taken to interpret differences between the two treatment positions. This volume difference is not explained by a difference in delineation, because the same oesophageal contouring method was used in prone and supine position. Thus, the volume change must be explained by the posture change in prone position. To our knowledge, this finding has not been reported elsewhere. Grantzau & Overgaard reported an association between radiotherapy and second oesophageal cancer, starting from 5 years post-radiation, with the RR increasing with time [44]. Decreasing the oesophageal dose may reduce secondary oesophageal cancer risk.

These dose reductions to the thyroid and oesophagus could be inherent to the prone position, but we hypothesize that these changes are more likely explained by to the different arm position. In the crawl position, the lymph node regions are moved away from the neck, thereby reducing doses to these organs.

One point of comment could be that the position of the arm next to the body might increase the dose to the arm and humeral head, because they are positioned closer to the affected breast. Irradiation of muscles and joints in this region could decrease mobility. Care also needs to be taken that the arm is not positioned in contact with the target regions, because this reduces possible beam directions for LNI and WBI. Moreover, it could eliminate build-up regions, increasing skin toxicity. Our study shows no significant difference in dose to the humeral head.

Prone radiotherapy for the breast improves cardiac dose in most patients, especially in large breasted patients [10–12]. We observed a slightly lower heart dose in prone position, but failed to show a significant difference between the two treatment positions. However, in this study, patients were small breasted, all with breast volumes < 1000 ml in prone position. Breast volume exceeded 750 ml in only 2 patients, and mean breast volume was 658 ml (range 292–892 ml, standard deviation = 237 ml). The UK HeartSpare study showed that breath-hold techniques are more efficient in lowering heart dose than prone position [16, 17]. However, it has been demonstrated that prone breath-hold for WBI without LNI is feasible and at least as efficient as supine breath-hold. Moreover, it is easy to implement since the irradiated breast does not move as much as in supine position [14, 15]. The feasibility of breath-hold for WB + LNI in prone crawl position merits further investigation.

Data on prone WB + LNI are sparse, and comparisons are not straightforward. Shin et al. [20] report lower doses to the heart and ipsilateral lung, but over half of their patients had right sided breast cancer. They also limited LNI to level III-IV. Kainz et al. [19] report on helical tomotherapy for WB + LNI, with heart, contralateral breast, ipsilateral lung, thyroid and oesophageal doses that are substantially higher than in our study. However, in addition to levels I-IV, they included the internal mammary nodes for LNI, which makes direct comparison unfair. Lastly, Gielda et al. [18] had a similar trial setup, with comparable ipsilateral lung dose, but we report lower heart and contralateral breast doses, with improved nodal coverage in our trial.

One of the weaknesses of this study is the small patient group. Undertaking a similar study on a larger patient group could be viewed as unethical, because maintaining an intra-patient comparison requires more patients to undergo a second CT scan without clinical benefit, and increased radiation exposure. We preferred to study a small patient group, and proceed to a clinical trial, as results were favourable in this group. Another weakness is the use of non-parametric testing of the dosimetric values because of the limited number of observations and the data not always being distributed normally. To reduce variability between patients in this small group, we excluded boost doses to the tumor bed from our analysis. It stands to reason that prone positioning will yield lower OAR doses than supine positioning, because this data already exists for WBI. However, as the boost dose was not analysed in our study, we can only make the assumption that OAR doses will be lower in prone crawl positioning. The non-parametric method uses value ranks rather than absolute values and the significance level could be less clear, as non-parametric tests are more conservative, meaning that achieving significance in these tests is harder. We chose to perform a one-sided test, because it is mathematically impossible to achieve significance using a 2-sided test with confidence level of 0.05 in a paired sample Wilcoxon test with five test subjects. Overall, individual patient results were in favour of prone positioning.

The exclusion of the internal mammary nodes from the nodal target volumes is debatable. Recent evidence shows improved disease outcomes, but these studies have some flaws which make generalisation difficult. Poortmans et al. [2] randomized between WBI or thoracic wall irradiation without LNI or with LNI to both the internal mammary nodes and medial supraclavicular nodes. It is unclear if the benefits in this trial relate to LNI to the internal mammary nodes specifically. Furthermore, breast cancer mortality was significantly reduced in the LNI group, but overall mortality reductions were not statistically significant (although P was only 0.06), indicating a possible treatment related mortality. Whelan et al. [3] also randomized between WBI with LNI to the internal mammary, supraclavicular and axillary nodes or WBI without LNI. They saw improved disease-free survival but no overall survival benefit for LNI. Furthermore, side-effects such as acute pneumonitis and lymphedema were increased in the LNI group. A trial by Thorsen et al. [46] treated right sided breast cancer patients with LNI to the internal mammary nodes and left sided breast cancer patients without LNI to the internal mammary nodes. Survival was improved in patients treated with internal mammary node irradiation, but results cannot be extrapolated to left sided breast cancer patients because of the increased toxicity risk due to radiation exposure of the heart in left sided breast cancer patients. Because of the unclear overall survival benefit and the higher treatment related toxicity and mortality risk, elective internal mammary node irradiation is not done routinely at our institution. We are confident, however, that improvements in radiation technique and patient positioning can decrease the dose to OARs and possibly decrease treatment related toxicities. The crawl couch allows beam incidences to treat the internal mammary nodes differently than in supine position. Given the recent evidence hinting at a beneficial effect of LNI to the internal mammary nodes, we are currently conducting a study where the internal mammary nodes are included in the nodal treatment volumes.

A last weakness is the absence of contouring guidelines for prone WB + LNI, which is why we extrapolated from existing guidelines. Our results hint at anatomic differences between the nodal target regions, which might have led to higher OAR doses. Developing an evidence based guideline for prone WB + LNI could decrease these doses, but this evidence will first have to be generated through further research.

## Conclusions

This pilot study demonstrates that prone crawl position for WB + LNI allows for good coverage of the nodal targets with better sparing of ipsilateral lung, thyroid, contralateral breast, contralateral lung and oesophagus compared to the supine position.

## Abbreviations

CBC: Contralateral breast cancer
CT: Computed tomography
CTV_LNI: Clinical target volume for lymph node irradiation
CTV_WBI: Clinical target volume for whole breast irradiation
D*n*: Minimal dose received by *n* % of a specified delineated volume
DVH: Dose-volume histogram
ESTRO: European Society for Radiotherapy & Oncology
GRATIS: George’s radiotherapy treatment design system
ICRU: International Commission on Radiation Units & Measurements
IMRT: Intensity modulated radiotherapy
LNI: Lymph node irradiation
MLC: Multi-leaf collimator
OAR: Organ at risk
PROCAB: Project on cancer of the breast
PTV_LNI: Planning target volume for lymph node irradiation
PTV_LNI_opt: Optimized planning target volume for lymph node irradiation
PTV_WBI: Planning target volume for whole breast irradiation
PTV_WBI_opt: Optimized planning target volume for lymph node irradiation
QUANTEC: Quantitative analyses of normal tissue effects in the clinic
RR: Relative risk
SPLC: Second primary lung cancer
VMAT: Volumetric modulated arc therapy
V*n*: Percentage of a specified delineated volume receiving at least *n* Gy
WB + LNI: Whole breast and lymph node irradiation
WBI: Whole breast irradiation

## References

[1] McGale P, Taylor C, Correa C, Cutter D, Duane F, Ewertz M, Gray R, Mannu G, Peto R, EBCTCG (2014). Effect of radiotherapy after mastectomy and axillary surgery on 10-year recurrence and 20-year breast cancer mortality: meta-analysis of individual patient data for 8135 women in 22 randomised trials. Lancet.

[2] Poortmans PM, Collette S, Kirkove C, Van Limbergen E, Budach V, Struikmans H, Collette L, Fourquet A, Maingon P, Valli M (2015). Internal Mammary and Medial Supraclavicular Irradiation in Breast Cancer. N Engl J Med.

[3] Whelan TJ, Olivotto IA, Parulekar WR, Ackerman I, Chua BH, Nabid A, Vallis KA, White JR, Rousseau P, Fortin A (2015). Regional Nodal Irradiation in Early-Stage Breast Cancer. N Engl J Med.

[4] Reznik J, Cicchetti MG, Degaspe B, Fitzgerald TJ (2005). Analysis of axillary coverage during tangential radiation therapy to the breast. Int J Radiat Oncol Biol Phys.

[5] Alco G, Igdem SI, Ercan T, Dincer M, Senturk R, Atilla S, Oral Zengin F, Okkan S (2010). Coverage of axillary lymph nodes with high tangential fields in breast radiotherapy. Br J Radiol.

[6] Jephcott CR, Tyldesley S, Swift CL (2004). Regional radiotherapy to axilla and supraclavicular fossa for adjuvant breast treatment: a comparison of four techniques. Int J Radiat Oncol Biol Phys.

[7] Viren T, Heikkila J, Myllyoja K, Koskela K, Lahtinen T, Seppala J (2015). Tangential volumetric modulated arc therapy technique for left-sided breast cancer radiotherapy. Radiat Oncol.

[8] Mulliez T, Speleers B, Madani I, De Gersem W, Veldeman L, De Neve W (2013). Whole breast radiotherapy in prone and supine position: is there a place for multi-beam IMRT?. Radiat Oncol.

[9] Dogan N, Cuttino L, Lloyd R, Bump EA, Arthur DW (2007). Optimized dose coverage of regional lymph nodes in breast cancer: the role of intensity-modulated radiotherapy. Int J Radiat Oncol Biol Phys.

[10] Sethi RA, No HS, Jozsef G, Ko JP, Formenti SC (2012). Comparison of three-dimensional versus intensity-modulated radiotherapy techniques to treat breast and axillary level III and supraclavicular nodes in a prone versus supine position. Radiother Oncol.

[11] Formenti SC, DeWyngaert JK, Jozsef G, Goldberg JD (2012). Prone vs supine positioning for breast cancer radiotherapy. JAMA.

[12] Kirby AM, Evans PM, Donovan EM, Convery HM, Haviland JS, Yarnold JR (2010). Prone versus supine positioning for whole and partial-breast radiotherapy: a comparison of non-target tissue dosimetry. Radiother Oncol.

[13] Lymberis SC, de Wyngaert JK, Parhar P, Chhabra AM, Fenton-Kerimian M, Chang J, Hochman T, Guth A, Roses D, Goldberg JD, Formenti SC (2012). Prospective assessment of optimal individual position (prone versus supine) for breast radiotherapy: volumetric and dosimetric correlations in 100 patients. Int J Radiat Oncol Biol Phys.

[14] Mulliez T, Van de Velde J, Veldeman L, De Gersem W, Vercauteren T, Speleers B, Degen H, Wouters J, Van Hoof T, van Greveling A (2015). Deep inspiration breath hold in the prone position retracts the heart from the breast and internal mammary lymph node region. Radiother Oncol.

[15] Mulliez T, Veldeman L, Speleers B, Mahjoubi K, Remouchamps V, Van Greveling A, Gilsoul M, Berwouts D, Lievens Y, Van den Broecke R, De Neve W (2015). Heart dose reduction by prone deep inspiration breath hold in left-sided breast irradiation. Radiother Oncol.

[16] Bartlett FR, Colgan RM, Carr K, Donovan EM, McNair HA, Locke I, Evans PM, Haviland JS, Yarnold JR, Kirby AM (2013). The UK HeartSpare Study: randomised evaluation of voluntary deep-inspiratory breath-hold in women undergoing breast radiotherapy. Radiother Oncol.

[17] Bartlett FR, Donovan EM, McNair HA, Corsini LA, Colgan RM, Evans PM, Maynard L, Griffin C, Haviland JS, Yarnold JR, Kirby AM (2017). The UK HeartSpare Study (Stage II): Multicentre Evaluation of a Voluntary Breath-hold Technique in Patients Receiving Breast Radiotherapy. Clin Oncol (R Coll Radiol).

[18] Gielda BT, Strauss JB, Marsh JC, Turian JV, Griem KL (2011). A Dosimetric Comparison Between the Supine and Prone Positions for Three-Field Intact Breast Radiotherapy. Am J Clin Oncol.

[19] Kainz K, White J, Chen GP, Hermand J, England M, Li XA (2012). Simultaneous irradiation of the breast and regional lymph nodes in prone position using helical tomotherapy. Br J Radiol.

[20] Shin SM, No HS, Vega RM, Fenton-Kerimian M, Maisonet O, Hitchen C, Keith DeWyngaert J, Formenti SC (2016). Breast, chest wall, and nodal irradiation with prone set-up: Results of a hypofractionated trial with a median follow-up of 35 months. Pract Radiat Oncol.

[21] Mason N, Macfarlane D, Guidi R, Owen R, Poulsen M (2012). A prone technique for treatment of the breast, supraclavicular and axillary nodes. J Med Imaging Radiat Oncol.

[22] Mulliez T, Veldeman L, van Greveling A, Speleers B, Sadeghi S, Berwouts D, Decoster F, Vercauteren T, De Gersem W, Van den Broecke R, De Neve W (2013). Hypofractionated whole breast irradiation for patients with large breasts: a randomized trial comparing prone and supine positions. Radiother Oncol.

[23] Offersen BV, Boersma LJ, Kirkove C, Hol S, Aznar MC, Biete Sola A, Kirova YM, Pignol JP, Remouchamps V, Verhoeven K (2015). ESTRO consensus guideline on target volume delineation for elective radiation therapy of early stage breast cancer. Radiother Oncol.

[24] Verhoeven K, Weltens C, Remouchamps V, Mahjoubi K, Veldeman L, Lengele B, Hortobagyi E, Kirkove C (2015). Vessel based delineation guidelines for the elective lymph node regions in breast cancer radiation therapy - PROCAB guidelines. Radiother Oncol.

[25] Verhoeven K, Weltens C, Remouchamps V, Mahjoubi K, Veldeman L, Lengele B, Hortobagyi E, Kirkove C (2016). Vessel based delineation guidelines for the elective lymph node regions in breast cancer radiation therapy - PROCAB guidelines. Radiother Oncol.

[26] Offersen BV, Boersma LJ, Kirkove C, Hol S, Aznar MC, Sola AB, Kirova YM, Pignol JP, Remouchamps V, Verhoeven K (2016). ESTRO consensus guideline on target volume delineation for elective radiation therapy of early stage breast cancer, version 1.1.. Radiother Oncol.

[27] Feng M, Moran JM, Koelling T, Chughtai A, Chan JL, Freedman L, Hayman JA, Jagsi R, Jolly S, Larouere J (2011). Development and validation of a heart atlas to study cardiac exposure to radiation following treatment for breast cancer. Int J Radiat Oncol Biol Phys.

[28] 3. Special Considerations Regarding Absorbed-Dose and Dose–Volume Prescribing and Reporting in IMRT. J ICRU. 2010;10:27–40. 10.1093/jicru/ndq008 24173325

[29] Merchant TE, McCormick B (1994). Prone position breast irradiation. Int J Radiat Oncol Biol Phys.

[30] Buijsen J, Jager JJ, Bovendeerd J, Voncken R, Borger JH, Boersma LJ, Murrer LH, Lambin P (2007). Prone breast irradiation for pendulous breasts. Radiother Oncol.

[31] Veldeman L, Schiettecatte K, De Sutter C, Monten C, van Greveling A, Berkovic P, Mulliez T, De Neve W (2016). The 2-Year Cosmetic Outcome of a Randomized Trial Comparing Prone and Supine Whole-Breast Irradiation in Large-Breasted Women. Int J Radiat Oncol Biol Phys.

[32] Alonso-Basanta M, Ko J, Babcock M, Dewyngaert JK, Formenti SC (2009). Coverage of axillary lymph nodes in supine vs. prone breast radiotherapy. Int J Radiat Oncol Biol Phys.

[33] Darby SC, McGale P, Taylor CW, Peto R (2005). Long-term mortality from heart disease and lung cancer after radiotherapy for early breast cancer: prospective cohort study of about 300,000 women in US SEER cancer registries. Lancet Oncol.

[34] Inskip PD, Stovall M, Flannery JT (1994). Lung cancer risk and radiation dose among women treated for breast cancer. J Natl Cancer Inst.

[35] Berrington de Gonzalez A, Curtis RE, Gilbert E, Berg CD, Smith SA, Stovall M, Ron E (2010). Second solid cancers after radiotherapy for breast cancer in SEER cancer registries. Br J Cancer.

[36] Grantzau T, Thomsen MS, Vaeth M, Overgaard J (2014). Risk of second primary lung cancer in women after radiotherapy for breast cancer. Radiother Oncol.

[37] Marks LB, Bentzen SM, Deasy JO, Kong FM, Bradley JD, Vogelius IS, El Naqa I, Hubbs JL, Lebesque JV, Timmerman RD (2010). Radiation dose-volume effects in the lung. Int J Radiat Oncol Biol Phys.

[38] Jo IY, Kay CS, Kim JY, Son SH, Kang YN, Jung JY, Kim KJ (2014). Significance of low-dose radiation distribution in development of radiation pneumonitis after helical-tomotherapy-based hypofractionated radiotherapy for pulmonary metastases. J Radiat Res.

[39] Stovall M, Smith SA, Langholz BM, Boice JD, Shore RE, Andersson M, Buchholz TA, Capanu M, Bernstein L, Lynch CF (2008). Dose to the contralateral breast from radiotherapy and risk of second primary breast cancer in the WECARE study. Int J Radiat Oncol Biol Phys.

[40] Boice JD, Harvey EB, Blettner M, Stovall M, Flannery JT (1992). Cancer in the contralateral breast after radiotherapy for breast cancer. N Engl J Med.

[41] Gao X, Fisher SG, Emami B (2003). Risk of second primary cancer in the contralateral breast in women treated for early-stage breast cancer: a population-based study. Int J Radiat Oncol Biol Phys.

[42] Finke I, Scholz-Kreisel P, Hennewig U, Blettner M, Spix C (2015). Radiotherapy and subsequent thyroid cancer in German childhood cancer survivors: a nested case–control study. Radiat Oncol.

[43] Sun LM, Lin CL, Liang JA, Huang WS, Kao CH (2015). Radiotherapy did not increase thyroid cancer risk among women with breast cancer: A nationwide population-based cohort study. Int J Cancer.

[44] Grantzau T, Overgaard J (2015). Risk of second non-breast cancer after radiotherapy for breast cancer: a systematic review and meta-analysis of 762,468 patients. Radiother Oncol.

[45] Vogelius IR, Bentzen SM, Maraldo MV, Petersen PM, Specht L (2011). Risk factors for radiation-induced hypothyroidism: a literature-based meta-analysis. Cancer.

[46] Thorsen LB, Offersen BV, Dano H, Berg M, Jensen I, Pedersen AN, Zimmermann SJ, Brodersen HJ, Overgaard M, Overgaard J (2016). DBCG-IMN: A Population-Based Cohort Study on the Effect of Internal Mammary Node Irradiation in Early Node-Positive Breast Cancer. J Clin Oncol.

